# Effects of a Novel Bradykinin B1 Receptor Antagonist and Angiotensin II Receptor Blockade on Experimental Myocardial Infarction in Rats

**DOI:** 10.1371/journal.pone.0051151

**Published:** 2012-12-07

**Authors:** Dongmei Wu, Xinchun Lin, Christian Bernloehr, Tobias Hildebrandt, Henri Doods

**Affiliations:** 1 Department of Research, Mount Sinai Medical Center, Miami Beach, Florida, United States of America; 2 Department of BIN Fusion Technology, WCU program, Chonbuk National University, Jeollabuk-do, Korea; 3 Boehringer Ingelheim Pharma GmbH & Co.KG, Biberach, Germany; Bristol Heart Institute, University of Bristol, United Kingdom

## Abstract

**Background:**

The aim of the present study was to evaluate the cardiovascular effects of the novel bradykinin B1 receptor antagonist BI-113823 following myocardial infarction (MI) and to determine whether B1 receptor blockade alters the cardiovascular effects of an angiotensin II type 1 (AT1) receptor antagonist after MI in rats.

**Methodology/Principal Findings:**

Sprague Dawley rats were subjected to permanent occlusion of the left descending coronary artery. Cardiovascular function was determined at 7 days post MI. Treatment with either B1 receptor antagonist (BI-113823) or AT1 receptor antagonist (irbesartan) alone or in combination improved post-MI cardiac function as evidenced by attenuation of elevated left ventricular end diastolic pressure (LVEDP); greater first derivative of left ventricular pressure (± dp/dt max), left ventricle ejection fraction, fractional shorting, and better wall motion; as we as reductions in post-MI up-regulation of matrix metalloproteinases 2 (MMP-2) and collagen III. In addition, the cardiac up-regulation of B1 receptor and AT1 receptor mRNA were markedly reduced in animals treated with BI 113823, although bradykinin B2 receptor and angiotensin 1 converting enzyme (ACE1) mRNA expression were not significantly affected by B1 receptor blockade.

**Conclusions/Significance:**

The present study demonstrates that treatment with the novel B1 receptor antagonist, BI-113823 improves post-MI cardiac function and does not influence the cardiovascular effects of AT1 receptor antagonist following MI.

## Introduction

Kinins are biologically active peptides that exert a broad spectrum of physiological effects, including vasodilation, smooth muscle contraction, inflammation, and pain induction [1]. The biological effects of kinins are mediated through the stimulation of bradykinin B1 and B2 receptors. The latter type is constitutively expressed and is activated by intact kinins, bradykinin, and kallidin. The B2 receptor is believed to play an important role in mediating the beneficial effects of angiotensin 1 converting enzyme (ACE) inhibitors used to treat cardiovascular diseases, but it is also involved in the acute phases of inflammation and of somatic and visceral pain [1]–[3]. Conversely, the B1 receptor is activated by the carboxypeptidase metabolites of kinins, des-Arg9-BK, and des- Arg10-kallidin. The B1 receptor is normally weakly expressed, but it is up regulated in the presence of cytokines and endotoxins or during tissue injury [1]–[3]. The B1 receptor participates in chronic inflammation and pain [2], [3]; thus, bradykinin B1 receptor antagonists are a potentially novel approach for treating these conditions.

Members of the kinin peptide family are important mediators of cardiovascular homeostasis. Bradykinin binding sites have been described in both myocytes and cardiac fibroblasts [4]. The importance of kinins in regulating cardiovascular physiology has been documented in B2 receptor knockout mice that develop hypertension and cardiac failure [5]. However, the role of the B1 receptor in heart has been controversial [6], [7]. Xu et al. suggested that the kinin B1 receptor is involved in the cardioprotective effect of ACE inhibitors and angiotensin receptor blockers in mice [7]. Conversely, other findings suggest that B1 receptor induction following tissue injury may be detrimental for cardiac function [8], [9]. B1 receptor deletion in mice protected from against cardiac ischemia-reperfusion injury [8], and following doxorubicin-induced cardiomyopathy [10]. Thus, selective B1 receptor inhibitors may have a favorable cardiovascular profile.

BI-113823 is a novel potent and selective B1 receptor antagonist that exhibits high affinity (Ki) for both human and rat B1 receptor (5.3 and 13.3 nM, respectively) [11]. BI-113823 inhibits the B1 receptor-cyclic adenosine monophosphate formation with a half maximal inhibitory concentration value of 19.1 nM, and it exerts analgesic properties in several animal models. It dose-dependently reversed the effects observed in Freund’s adjuvant (CFA) model, the weight bearing deficit in the monoiodoacetate model, and mechanical hyperalgesia in the carrageenan model [11]. The compound has no affinity for the B2 receptor (IC50>10.000 nM) and proved to be highly selective versus a large panel other receptors/enzymes or channels. Especially, we examined whether BI 113823 directly interacts with angiotensin receptor or has an effects on blood pressure in rata. BI 113823 is devoid of an interaction with the angiotensin receptor and does not influence blood pressure in conscious rats in doses exceeding those used in the present study. Moreover, BI 113823 does not interfere with the blood pressure lowering effects of lisinopril in spontaneoulsly hypertensive rats following a 2 weeks treatment period. The goal of this present study was to evaluate the effects of BI-113823 following myocardial infarction (MI) in rats and to determinate whether B1 receptor blockade with BI-113823 affects the cardiovascular effects of an angiotensin II type 1 (AT1) receptor antagonist following MI in rats.

## Methods

### Animals

All animal studies were approved by the Institutional Animal Care and Use Committee at Mount Sinai Medical Center and complied with the Animal Welfare Act. Sprague Dawley rats weighing 275–325 g were used in all experiments. The rats were housed in a temperature-controlled room with a 12∶12-h light-dark cycle and were given standard chow and tap water. All animals were observed daily for general health, and all invasive procedures were performed under sterile conditions.

### In vivo Myocardial Ischemia Model

Rats were anaesthetized with intramuscular (i.m.) ketamine (60 mg/kg) plus xylazine (10 mg/kg, i.m.). The animals were intubated and ventilated with room air with a rodent respirator (Columbus Instruments, Columbus, OH, USA). A left-sided thoracotomy was performed at the level of the fourth intercostal space. The left anterior descending coronary artery was ligated near its origin with a 6-0 silk suture. The incisions were then closed in layers with 5.0 Vicryl suture, and the animals were gradually weaned from the respirator.

Beginning 24 hours after MI induction, animals received daily treatments of vehicle (0.5% Natrosol +0.01% TWEEN80), B1 receptor antagonist (BI 113823, 30 mg/kg, p.o.), AT1 antagonist (irbesartan, 30 mg/kg/day, p.o.), or both BI-113823 and irbesartan. The doses were selected based on the dosages used as effective analgesic drugs in clinical and laboratory studies [11], [12]. The experiment was terminated 7 days after MI induction.

### Echocardiography

Echocardiography (ECG) was performed in anesthetized animals 7 days after MI with a Hewlett-Packard echocardiographic system SONOS 2000 with a 7.5/5.5 MHz transducer. In each animal, a two-dimensional short-axis view was taken at the mid-papillary muscle level to obtain left ventricular (LV) ejection fraction (EF). Linear dimensions were measured from two-dimensionally guided M-mode tracing, and fractional shortening (FS) was obtained. An ECG tracing was recorded simultaneously with the echocardiogram. Wall motion score index (WMSI) was the sum of wall motion scores divided by the number of visualized segments. In this scoring system, higher scores indicate more severe wall motion abnormalities: 1 =  normal, 2 =  hypokinesis, 3 =  akinesis, 4 =  dyskinesis, 5 =  aneurysm [13]. All measurements were repeated three times, and the results represent the average of these measurements.

**Table 1:** Ratios of heart weight to body weight (HW/BW) and lung weight to body weight (LW/BW) and infarct size 7 days after MI. Values are mean ± SD, n = 8.

	Sham	Vehicle	BI-1	IRB	IRB+BI-1
**HW/BW** (mg/gram)	3.37±0.09	3.47±0.10	3.40±0.06	3.42±0.07	3.50±0.10
**LW/BW** (mg/gram)	4.31±0.13	5.04±0.08#	4.66±0.08*	4.71±0.10*	4.88±0.16#
**Infarct size** (% of LV)	N/A	31.5±2.12	32.4±1.49	30.4±1.31	33.1±1.69

Sham, sham-operated rats without coronary occlusion; Vehicle, without any medication; BI1, treated with B1 receptor antagonist (BI 113823); IRB, treated with AT1 receptor antagonist (irbesartan); BI1+IRB, treated with both BI 113823 and irbesartan.

**Table 2:** Hemodynamic parameter changes in rats 7 days after MI. All values are mean ± SD, n = 8.

	Sham	Vehicle	BI-1	IRB	IRB+BI-1
**MBP (mmHg)**	113.2±5.1	99.3±3.0	106.7±4.7	103.8±3.1	99.4±5.6
**HR (beats/min)**	231±12	235±13	227±11	236±13	220±10
**LVP (mmHg)**	110.5±10.5	79.9±6.2#	91.6±7.5*	75.3±10.1#	86.7±4.7*
**LVEDP (mmHg)**	14.8±0.2	25.5±2.5#	19.4±1.8#*	17.1±1.1*	13.5±1.2*
**± dp/dt max (mmHg/sec)**	1984±114	1169±198#	1598±121#*	1165±223#	1545±125#*

MBP, mean arterial blood pressure; HR, heart rate; LVP, left ventricular systolic pressure; LVEDP, left ventricle end-diastolic pressure; ± dp/dt max, first derivative of left ventricular pressure.

* p<0.05 vs. vehicle;

#p<0.05 vs. sham.

### Hemodynamic Assessment and Tissue Harvest

Animals were anesthetized with ketamine (60 mg/kg, i.m.) plus xylazine (10 mg/kg, i.m.). The left external jugular vein was cannulated for right atrial pressure measurement. A catheter was inserted into the right common carotid artery for quantifying arterial blood pressure with a Powerlab data acquisition system (ADInstruments Inc., CO). Heart rate was derived from the blood pressure signal. After arterial blood pressure was measured, the catheter was introduced into the left ventricle through the right carotid artery to monitor left ventricular pressure (LVP) and its first derivative (± dp/dp max).

At the end of experiment, hearts and lungs from MI and sham-operated rats were collected and weighed. Left ventricles were macroscopically separated and cut from the apex to base into four equal sections. The cut surface of each section was scanned for densitometry. The infarcted and non-infarcted left ventricle areas were separated and snap-frozen until later analysis.

### Reverse-transcription Polymerase Chain Reaction (RT-PCR)

B1 receptor, B2 receptor, AT1 receptor, and ACE1 transcript levels were assessed by quantitative RT-PCR. Total RNA was extracted from both the non-infarcted and infarcted areas of left ventricle. cDNA synthesis was performed with a High Capacity cDNA Reverse Transcription kit (Applied Biosystems, Carlsbad, CA, USA) on 1 µg RNA. For each RT-PCR reaction, 20ng cDNA was used in a QuantiFast Probe PCR kit (Qiagen). Primers were targeted against rat B1 receptor (Bdkrb1) (Applied Biosystems Gene Expression Assay Rn02064589_s1), rat B2 receptor (Bdkrb2) (Applied Biosystems Gene Expression Assay Rn00597384_m1), rat angiotensin II receptor, type 1a (Agtr1a; AT1) (forward: cacccgatcaccgatcac; reverse: cagccattttataccaatctctca; UPL probe set 53, cat. no. 04688503001, Roche, Basel, Switzerland) and rat angiotensin I converting enzyme (peptidyl-dipeptidase A) 1 (ACE1) (forward: ggttttcatgaggctattgga; reverse: ttagaaagttgatgtcatgctcgt; UPL probe set 21, cat. no. 04686942001, Roche). AT1 and ACE1 primers were designed using ProbeFinder v2.45 for rat (Roche). All data were normalized against RNA polymerase 2 (forward: GCAGGCGAGAGCGTTGAG; reverse: CATTGGTATAATCAAAACGGAACTTC; probe: CTGGCTACACTTAAGCCTTCTAATAAAGC, FAM/TAMRA-labeled).

Amplifications were performed in triplicate using an ABI Prism 7900HT Sequence Detection System (Applied Biosystems).

### Western Blot Analysis

Western blotting was performed as we described previously [14]. Briefly, left ventricle protein extracts were electrophoresed on NuPAGE 4–12% Bis-Tris gels (Invitrogen, Carlsbad, CA) and transferred onto nitrocellulose membranes (Invitrogen). The blots were then incubated in 5% nonfat dry milk in Tris-buffered saline (TBS) for 2 hours at room temperature, and then incubated with primary antibody for 1 hour at room temperature: collagen III (1∶1000) (Sigma), matrix metalloproteinases 2 (MMP-2) (1∶1000) and GAPDH (1∶3000) (both from Santa Cruz Biotechnology, Santa Cruz, CA). The blots were rinsed with TBS and incubated with horse radish peroxidase-conjugated donkey anti-rabbit or goat anti-mouse IgG secondary antibody for 2 hours. Immunoreactivity was detected using enhanced chemiluminescence autoradiography (ECL kit; Amersham, Piscataway, NJ). The signals were scanned with a color image scanner and densometrically quantified with ImageJ software (National Institutes of Health, Bethesda, MD, USA).

**Figure 1:**
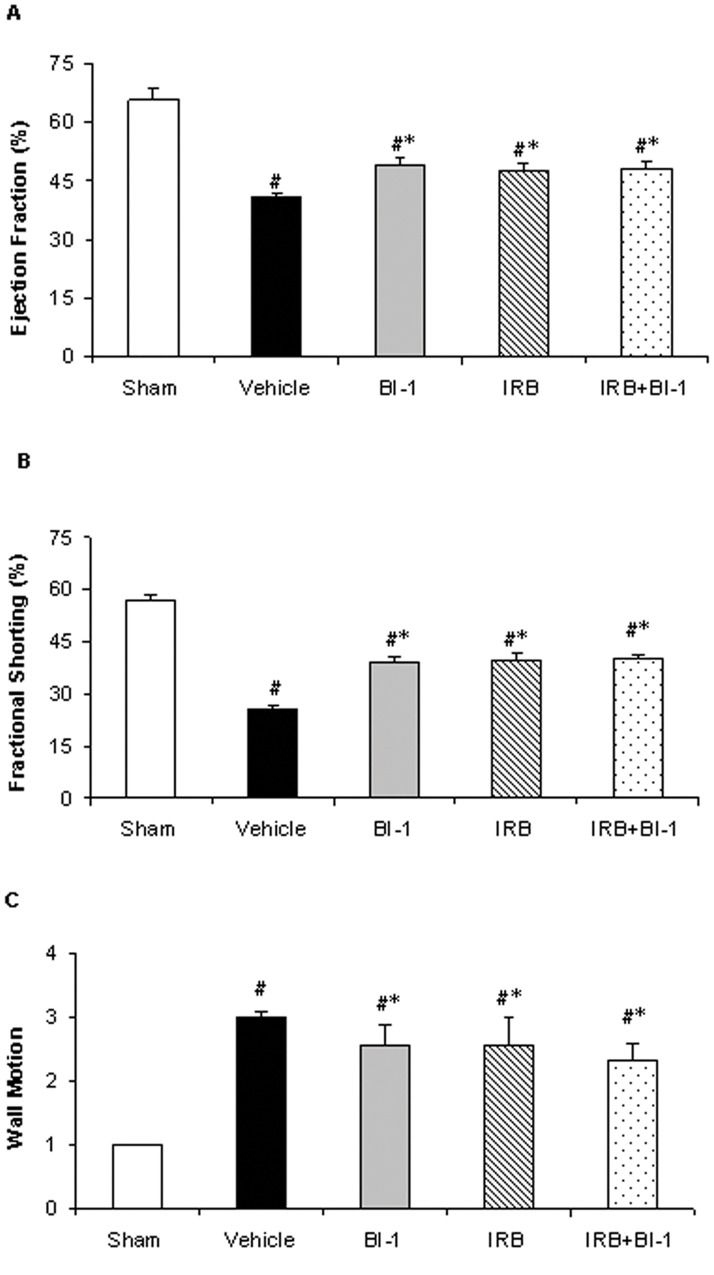
Echocardiographic measurements of left ventricular function following myocardial infarction in rat. *A):* left ventricular ejection fraction (EF), *B):* fractional shorting (FS), and *C):* wall motion. All values are mean ± SD, n = 8. *p<0.05 vs. vehicle control; #p<0.05 vs. sham. Sham, sham-operated rats without coronary occlusion; Vehicle, without any medication; BI1, treated with B1 receptor antagonist (BI 113823); IRB, treated with AT1 receptor antagonist (irbesartan); BI1+IRB, treated with BI 113823 and irbesartan.

**Figure 2:**
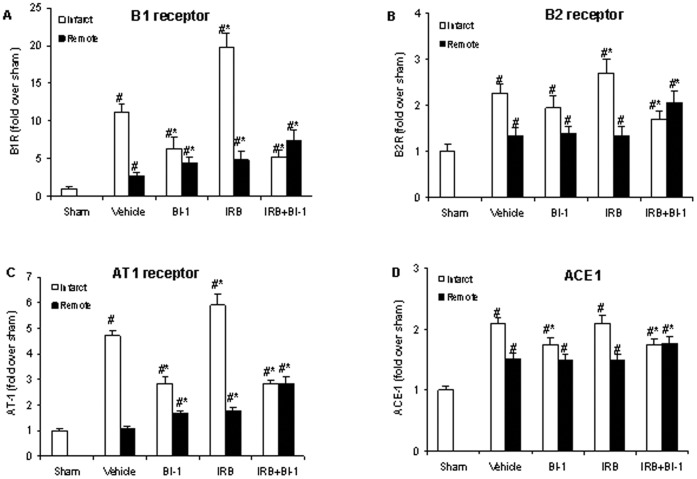
Reverse-transcription polymerase chain reaction (RT-PCR) data for endogenous expression of *A):* B1 receptor, *B):* B2 receptor, *C):* AT1 receptor, and *D):* ACE1 mRNA in rat left ventricle after MI. Sham levels were set at 1, and all other values were normalized to Sham. All values are mean ± SD, n = 6. *p<0.05 vs. vehicle control; #p<0.05 vs. sham.

**Figure 3:**
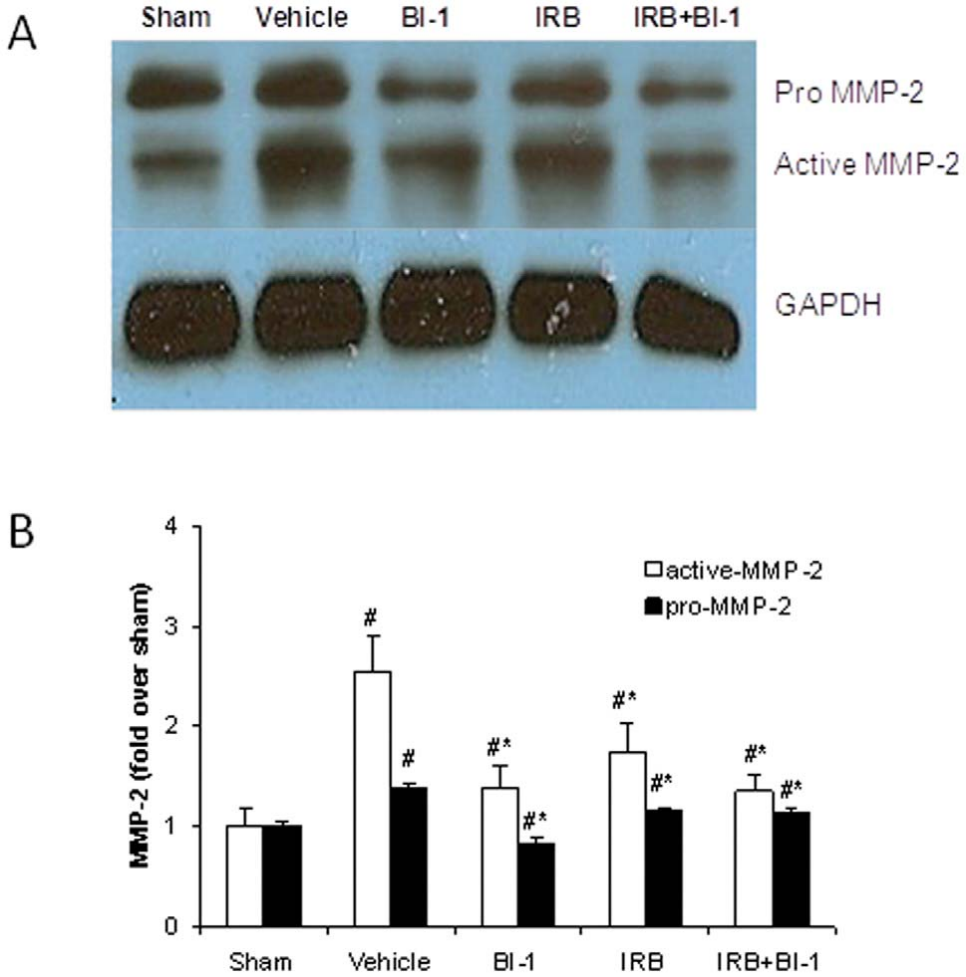
Protein expression of active MMP-2 and pro MMP-2 in rat left ventricle after MI. *A):* Immunoblots for pro MMP-2, active MMP-2, and GAPDH in left ventricle (remote area). *B):* mean densitometric analysis of pro and active MMP-2. Sham levels were set at 1, and all other values were normalized to Sham. All values are mean ± SD, n = 6. *p<0.05 vs. vehicle control; #p<0.05 vs. sham.

**Figure 4:**
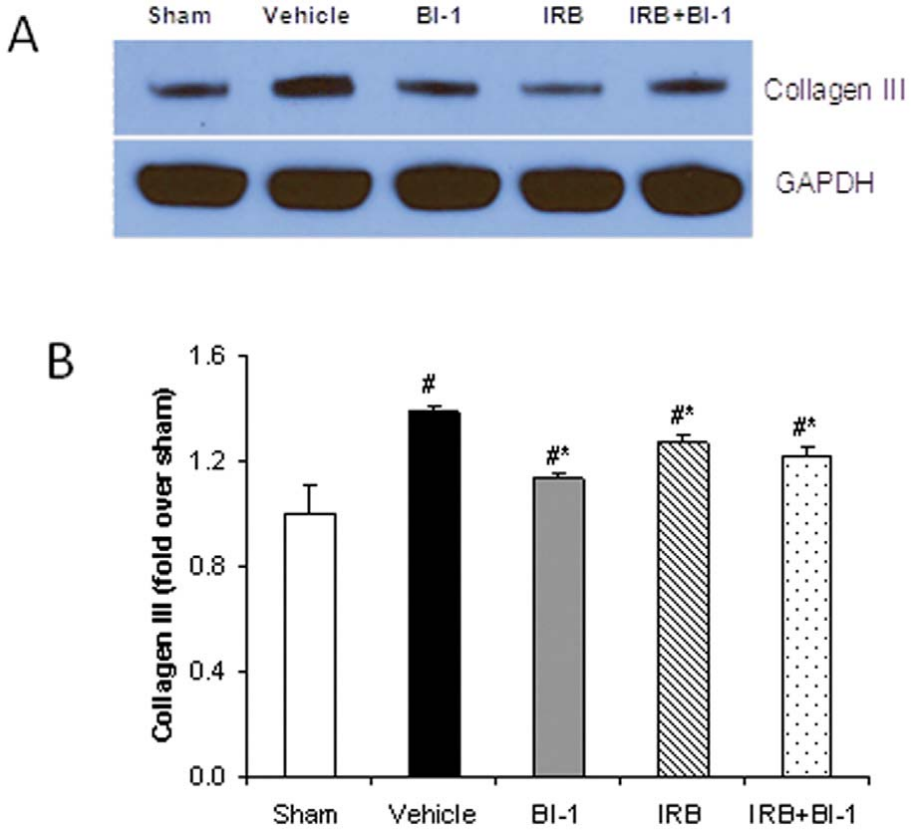
Protein expression of collagen III in rat left ventricle after MI. *A):* Immunoblots for collagen III and GAPDH in left ventricle (remote area). *B):* mean densitometric analysis of collagen III. Sham levels were set at 1, and all other values were normalized to Sham. All values are mean ± SD, n = 6. *p<0.05 vs. vehicle control; #p<0.05 vs. sham.

### Statistical Analysis

All results are presented as mean ± standard deviation (SD). Data were analyzed using two-sided analysis of variance (ANOVA) followed by post hoc intergroup comparisons using Student’s t-tests with Bonferroni corrections. P values <0.05 were considered statistically significant.

## Results

Eight sham-operated rats and 32 rats that initially survived acute MI induced by left coronary artery occlusion were weaned off the ventilator, randomly assigned to study groups, and survived the entire experimental protocol. At 7 days after MI, there was no significant difference in infarct size among study groups (table 1). The heart weight to body weight ratio (HW/BW) and lung weight to body weight ratio (LW/BW) are shown in table 1. The HW/BW ratio was not significantly increased 7 days after MI compared to sham animals. However, the LW/BW ratio was significantly increased 7 days after MI in vehicle-treated control rats compared to sham animals. This increase was significantly attenuated in animals treated with irbesartan or BI-113823 alone or a combination of irbesartan and BI-113823 (P>0.05).

Hemodynamic parameters are shown in table 2. There were no significant changes in mean blood pressure or heart rate in any of the study groups. In vehicle-treated control animals, MI resulted in decreased left ventricle systolic pressure (LVP) and ±dp/dt max and elevated left ventricle end-diastolic pressure (LVEDP). LVP was not significantly different among treatment groups compared to the vehicle control group. The ± dp/dt max was improved in animals that received BI-113823 or a combination of irbesartan and BI-113823, but not in animals that received irbesartan alone. Post-MI LVEDP elevation was significantly attenuated in all treatment groups compared to vehicle control (table 2).

Echocardiography analysis shows that MI resulted in impaired left ventricular function evidence by reduced ejection fraction and fractional shorting and impaired wall motion in vehicle-treated control animals. All three treatment groups exhibited improvements in all three parameters (Fig. 1).

### mRNA Expression Following MI

MI markedly increased cardiac mRNA expression of B1, B2, and AT1 receptors and ACE1 in the left ventricle, and this up-regulation was greater in infarcted regions [B1 receptor (11.1x), B2 receptor (2.3x), AT1 receptor (4.7x), and ACE1 (2.1x)], compared to remote regions [B1 receptor (2.7x), B2 receptor (1.4x), AT1 receptor (1.7x), and ACE1 (1.5x)] (Fig. 2). B1 and AT1 receptor mRNA upregulation were markedly reduced in animals treated with BI 113823, but B2 receptor and ACE1 mRNA expression were not significantly affected by B1 receptor blockade compared to vehicles controls. Treatment with the AT1 receptor antagonist irbesartan increased mRNA expression of B1, B2, and AT1 receptors but did not affect ACE1 expression compared to vehicle-treated controls. These up-regulations were markedly reduced in animals that received both BI-113823 and irbesartan, but the combination treatment did not affect ACE1 expression.

### Western Blot Analysis

MMP-2 cardiac expression was significantly increased following MI, and this increase was more pronounced with active MMP-2 compared to pro MMP-2 (Fig. 3). Cardiac collagen III was also increased following MI (Fig. 4). The up-regulation of MMP-2 and collagen following MI was attenuated by treatment with BI-113823 or irbesartan alone or given together (Fig. 3–4).

## Discussion

This study evaluated the cardiovascular effect of the newly developed novel B1 receptor antagonist BI-113823 and the AT1 receptor antagonist irbesartan alone or in combination following acute MI in rats. Cardiac function was impaired 7 days after MI induction but was attenuated by all three treatment paradigms.

Kinins are released directly from the myocardium during MI, and this can continue for several weeks and perpetuate further ischemic damage [1], [15]. The native peptide bradykinin is a potent vasodilator peptide and B2 receptor agonist that possesses various cardioprotective actions that are presumed to result from nitric oxide and/or prostacyclin production [16]. In contrast to B2 receptor-mediated relaxation downstream of bradykinin, the carboxypeptidase metabolite of bradykinin, des-Arg9-BK, is a B1 receptor agonist that causes vasoconstriction in various vascular beds, including pig coronary and pulmonary artery, rabbit basilar and carotid artery, rat portal vein, and isolated perfused rat kidneys [17]–[20]. Thus, the vascular function of B1 receptor is often opposed to that of the B2 receptor.

Previous studies have reported up-regulation of both B2 and B1 receptors hours and days after MI [1], [21]. In the present study, we found marked up-regulation of mRNA for cardiac B1 receptor, B2 receptor, AT1 receptor, and ACE1 after MI, and values were higher in infarcted left ventricle regions compared to remote regions. Treatment with B1 receptor antagonist BI-113823 markedly attenuated B1 and AT1 receptor up-regulation but had minimal effects on B2 receptor and ACE1 expression, suggesting that B2 and B1 receptors play different cardiovascular roles. Studies have reported the contribution of B2 receptor to the cardioprotective effect of bradykinin in cardiac disease models [22], [23]. Mice with targeted B2 receptor gene disruption develop hypertension, cardiac hypertrophy, cardiac fibrosis, and cardiac failure [22]. Moreover, pharmacological blockade of B2 receptor reverses the anti-hypertrophic action of bradykinin in rats [23]. These studies suggest that bradykinin is cardioprotective via B2 receptor activation, which attenuates myocardial damage. In contrast, others have reported that B1 receptor activation is associated with detrimental effects in various organ systems. B1 receptor deletion or blockade protects mice from focal brain injury by reducing blood-brain barrier leakage and inflammation, can attenuate cardiac inflammation and fibrosis during experimental diabetic cardiomyopathy, and it can ameliorate cardiac and renal ischemia-reperfusion injury and glomerulonephritis [8], [10], [24]–[27]. In addition, B2 receptor activation activates endothelial nitric oxide synthase (eNOS) and simulates the release of nitric oxide (NO), prostacyclin, and kinins to inhibit vascular smooth muscle growth and neointima formation, which may inhibit atherosclerosis development; conversely, B1 receptor activation, results in a prolonged high output of NO by inducible nitric oxide synthase (iNOS) and may have deleterious effects [6]. Collectively, the evidence suggests that the B1 receptor mediates cardiac inflammation and fibrosis and may be detrimental to cardiac function.

Ventricular remodeling and cardiac fibrosis after MI are associated with up-regulation of MMPs and fibrillar collagen, which adversely affect cardiac function [28], [29]. In the present study, B1 receptor antagonist treatment attenuated increased cardiac expression of MMP-2 and collagen III after MI and prevented increased lung mass, resulting in improved cardiac function. B1 receptor and MMP synthesis are both regulated by a number of cytokines and growth factors, including tumor necrosis factor-α, interleukin-β, and transforming growth factor-β [30], [31]. Thus, our data support the hypothesis that B1 receptor up-regulation after MI may contribute to cardiac inflammation and fibrosis and cardiac dysfunction.

The beneficial effects of ACE inhibitors are attributed to reduced angiotensin II generation and bradykinin degradation [32]. ACE inhibitors enhance B2 and B1 receptor signaling and augment NO production. Greater B2 receptor signaling activates eNOS, yielding a short burst of NO; while activation of B1 receptor results in prolonged NO output by iNOS [32], [33]. Studies have shown that the cardioprotective effects of ACE inhibitors are reduced by co-treatment with a B2 receptor antagonist [34]. Unlike ACE inhibitors, AT1 receptor antagonists do not directly interfere with bradykinin degradation, and the cardioprotective effects of an AT1 receptor antagonist were reduced in B2 receptor knockout mice, further suggesting a cardioprotective role of B2 receptors [35]. It is not clear whether B1 receptor blockade affects the beneficial cardiovascular effects of AT1 antagonist after MI. To date, only one report examined pharmacological inhibition of both B1 receptor (with B9958, 0.1 mg/kg/48 h/s.c.) and AT1 following acute MI, and the authors suggested that B1 receptor inhibition attenuates the protective effect of AT1 receptor inhibition after MI; however, this conclusion was only based on the observation that animals that received both AT1 receptor antagonist and B1 receptor antagonist had lower hemodynamics, which were measured in “open-chest animals” [36]. The same group reported improved cardiac function in animals with B1 receptor deletion [10]. In contrast, we found that B1 receptor blockade neither reduced nor potentiated the cardiovascular effects of AT1 receptor antagonist after MI. All three treatment paradigms improved post-MI cardiac function as measured by cardiac catheterization and ECHO analysis and attenuated the post MI upregulation of MMPs and fibrillar collagen. It is worth mentioning that the discrepancy between these studies could be attributable to the properties of the two different B1 receptor antagonists that were used. In the present study, we used the novel B1 receptor antagonist BI 113823, which has 13-nM affinity for the rat B1 receptor [11]. To our knowledge, the affinity of B9958 for the rat B1 receptor has not been described. This is important because it is known that several B1 receptor antagonists exhibit species selectivity. Although B9958 is a potent antagonist for human and rabbit B1 receptors [37], the structural analogs of B9958 have much lower affinity for the murine B1 receptor [38]. In addition, it is unclear whether the plasma levels achieved with 0.1 mg/kg/48h/s.c. B9985 are high enough to significantly occupy the B1 receptor and/or exert a pharmacological effect. Given that aminopeptidases can limit in vivo potency and exposure [37], it is important to know the plasma levels achieved by Tschöpe et al. [36]. We used an oral dose of BI 113823 (30 mg/kg b.i.d.) that was 10-fold higher than the dose required to elicit an analgesic effect in the CFA inflammatory pain model. Moreover, plasma sample determination confirmed that we achieved plasma levels over 24 h that exceeded the Ki value of BI 113823 for the rat receptor (data not shown). Finally, BI 113823 was assessed in a large receptor/channel screen, which showed that it is highly selective for the B1 receptor and suggested that it does not cause non specific effects. Similar data have not been reported for B9958. Accordingly, pharmacokinetic profile and rat receptor affinity differences might explain the discrepancies between these two studies.

Kinins have also been shown to exert an important role in post ischemic angiogenesis and regeneration [39], [40]. Kinins can stimulate neovascularization acting through signaling of both B1 and B2 receptors [41]–[43]. Studies have shown that kinins exert proliferative effects on endothelial cells via an IP3K-Akt-NO mediated mechanism independent of VEGF [41]–[45]. In a rat mesenteric arteriogenesis model, Stone et al. showed that kinins lead to a B2 receptor-dependent proliferation of endothelial cells, migration of pericytes and vascular smooth muscle cells, which contributed to the neoangiogenesis. Furthermore, the authors also demonstrate that kallikrein directly stimulates the production of MMPs, which are proangiogenic enzymes [40], [46]. Recent studies have further demonstrated that kinins are involved in B2 receptor-dependent recruitment of circulating endothelial progenitor cells (EPCs), which contribute to neovascularization and endothelial repair [43], [47]. In addition, Spillmann et al. showed that adenoviral transfer of the human tissue kallikrein gene into the peri-infarct myocardium in mice results in activation of the KKS, stimulation of the homing of cardiac progenitor cells and suppression of apoptosis, leading to stronger neovascularization [48]. On the other hand, the mechanism involved in B1 receptor-dependent neovascularization has not been well characterized, which needs to be further elucidated in further works.

Collectively, most of the research data show a pivotal role for the B2 recptor in cardio protective effects. The B2 receptor exerts anti-inflammatory, antiapoptotic and antifibrotic effects, leading to an ameliorated myocardial inflammation, remodeling and function in post myocardial infarction [40]. While the B1 receptor seems to have mostly detrimental effects in the early stage of MI, when strong activation of the immune system may lead to increased inflammation and impaired cardiac function [40].

In summary, B1 receptor activity seems to be involved in the pathogenesis of chronic pain and inflammation. Thus, targeting bradykinin B1 receptors with antagonists offer a potentially novel approach for treating chronic pain and inflammation. The present study shows that treatment with the novel B1 receptor antagonist BI-113823 improves cardiac function following experimental MI through attenuation of MMP-2 and fibrillar collagen expression and does not affect the cardiovascular effects of AT1 receptor antagonist. Collectively, these data indicate that the newly developed novel B1 receptor antagonist BI-113823 has a favorable cardiovascular profile.
